# Ruptured infected external iliac artery aneurysm following total hip arthroplasty successfully treated with endovascular stent graft placement: A case report

**DOI:** 10.1016/j.jccase.2026.01.012

**Published:** 2026-02-09

**Authors:** Takeshi Okura, Toshitaka Okabe, Naoei Isomura, Masahiko Ochiai

**Affiliations:** Division of Cardiology, Showa Medical University Northern Yokohama Hospital, Yokohama, Kanagawa, Japan

**Keywords:** Hip arthroplasty, Infected aneurysm, Stent graft

## Abstract

Although rare, infected aneurysm formation following hip arthroplasty is a serious complication that can be life-threatening. The current patient was a 58-year-old woman who had undergone left hip arthroplasty 7 years earlier and bilateral hip arthroplasty 3 years earlier. She presented with pain in her left lower leg. She was diagnosed with left hip joint dislocation and admitted for revision surgery. After admission blood tests revealed elevated inflammatory markers and progressive anemia, and worsening swelling and pain in the left lower leg. Computed tomography revealed a ruptured aneurysm of the left external iliac artery. It was hypothesized that a periprosthetic joint infection had contributed to the development and rupture of the aneurysm. She underwent endovascular therapy using a stent graft. The infected artificial hip joint was then surgically removed, and antimicrobial therapy was administered. Her condition improved after treatment. One of the major concerns with stent graft treatment in infected aneurysms is the persistence of infected tissue. While endovascular therapy can be effective, debridement of infected lesions remains the standard approach for long-term infection control. We suggest that stent graft placement may be a viable treatment option in selected cases.

**Learning objective:**

Infected aneurysm formation after hip arthroplasty is rare. In the current emergent case of aneurysmal rupture, considering the patient's overall condition, we initially opted for endovascular stent graft placement. In the event that the infection was not controlled, secondary surgical replacement with an artificial graft was planned. The case demonstrates the potential utility of such a staged treatment strategy.

## Introduction

Aneurysm formation in the chronic phase after hip arthroplasty is rare, but several cases have been reported [1]. Factors contributing to aneurysm formation include mechanical stress on blood vessels caused by the implants, and deep infections extending into the pelvis, where inflammation of the surrounding tissues can compromise the integrity of the arterial wall [2], [3]. Ruptured iliac artery aneurysms are associated with high mortality, and require immediate surgical intervention [4]. Although endovascular therapy (EVT) for infected aneurysms is uncommon, herein we report a life-saving approach combining EVT and antimicrobial agents.

## Case report

The patient was a 58-year-old woman who presented with pain in her left lower leg.

She had undergone a total hip arthroplasty (THA) 7 years prior, followed by revision surgery for prosthetic hip dislocation 3 years after that surgery. Because X-ray revealed a left hip joint dislocation, the orthopedic team had planned hospital admission for revision surgery. However, after experiencing a fall the patient developed impaired mobility and was admitted to the hospital on an emergency basis. Other relevant medical history included primary biliary cholangitis and rheumatoid arthritis, for which she had been taking methotrexate (4 mg/week as) as an immunosuppressive drug for three years prior to admission. Anticoagulants were administered because computed tomography (CT) on admission revealed deep vein thrombosis.

**Fig. 1:**
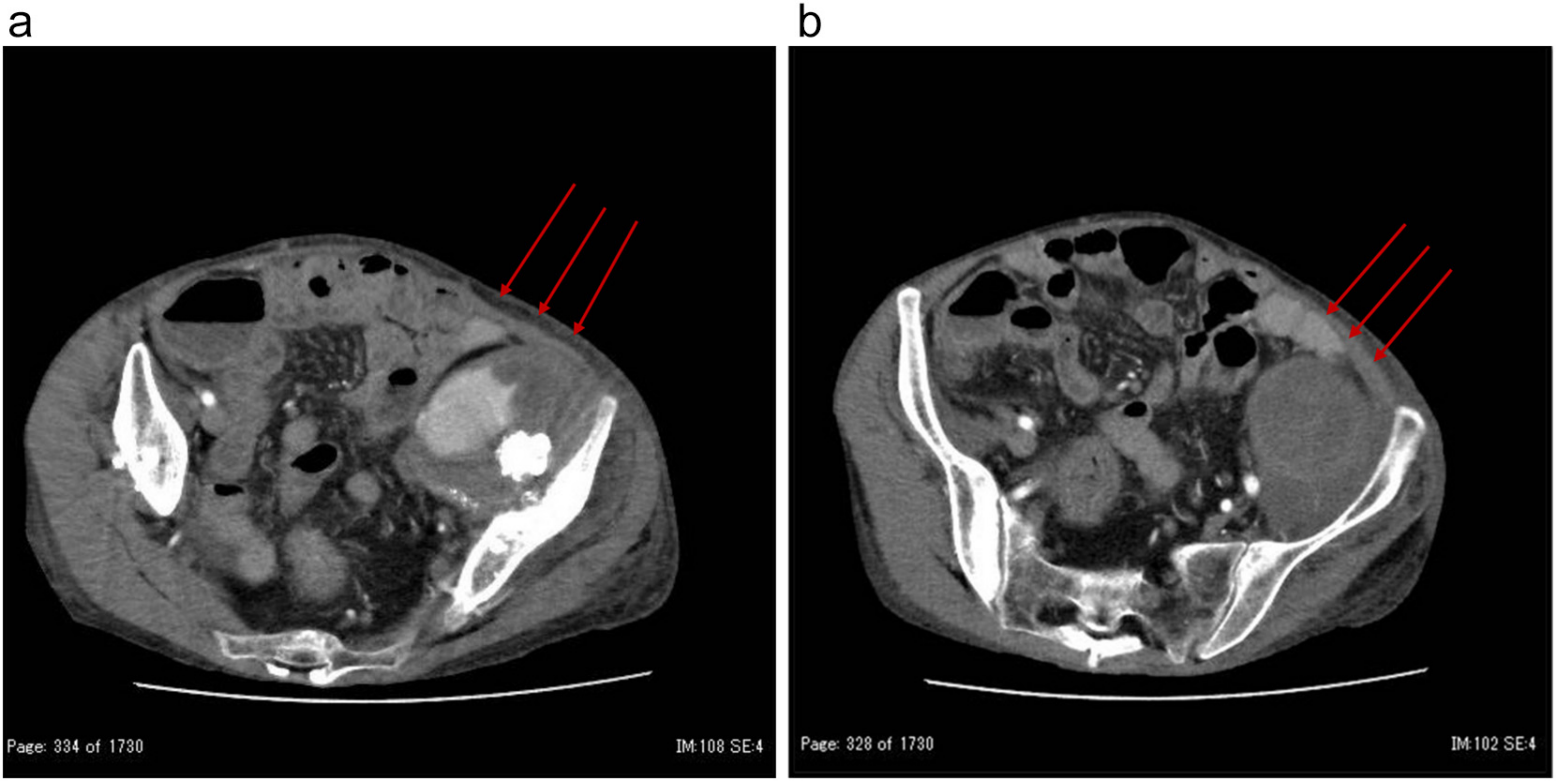
(A, B) Computed tomography revealed suspected rupture of the external iliac artery.

Two weeks after admission, laboratory data revealed elevated inflammatory markers and progressive anemia, in conjunction with worsening swelling and pain in the left lower leg, and notably without back pain or neurological symptoms. Repeat CT revealed suspected rupture of the external iliac artery (EIA) that had not been identified on initial CT at admission (Fig. 1A, B). It was suspected that a prosthetic joint infection had developed due to immunosuppressive therapy, subsequently leading to the formation and rupture of the EIA aneurysm. After consultation with the vascular and endovascular team, EVT was selected as the initial management strategy. Peripheral angiography was performed under general anesthesia because severe leg pain prevented her from maintaining a supine position.

EVT was performed via the right femoral artery approach. A 7 French, 45-cm guide catheter (Destination, Terumo, Tokyo, Japan) was inserted into the right common femoral artery. Intravascular ultrasound and a 0.014-inch, 300-cm guide wire (Vassalo GT NS1, Cordis, Miami Lakes, FL, USA) toward the left superficial femoral artery were used to identify the bleeding site in the EIA (Video 1). A 7 × 100-mm VIABAHN endoprosthesis (W. L. Gore & Associates, Flagstaff, AZ, USA) was implanted, and a 5-mm non-compliant balloon was used for post-dilation. After post-dilation, final angiography indicated that the ruptured aneurysm was successfully excluded without any major complications (Video 2).

**Fig. 2:**
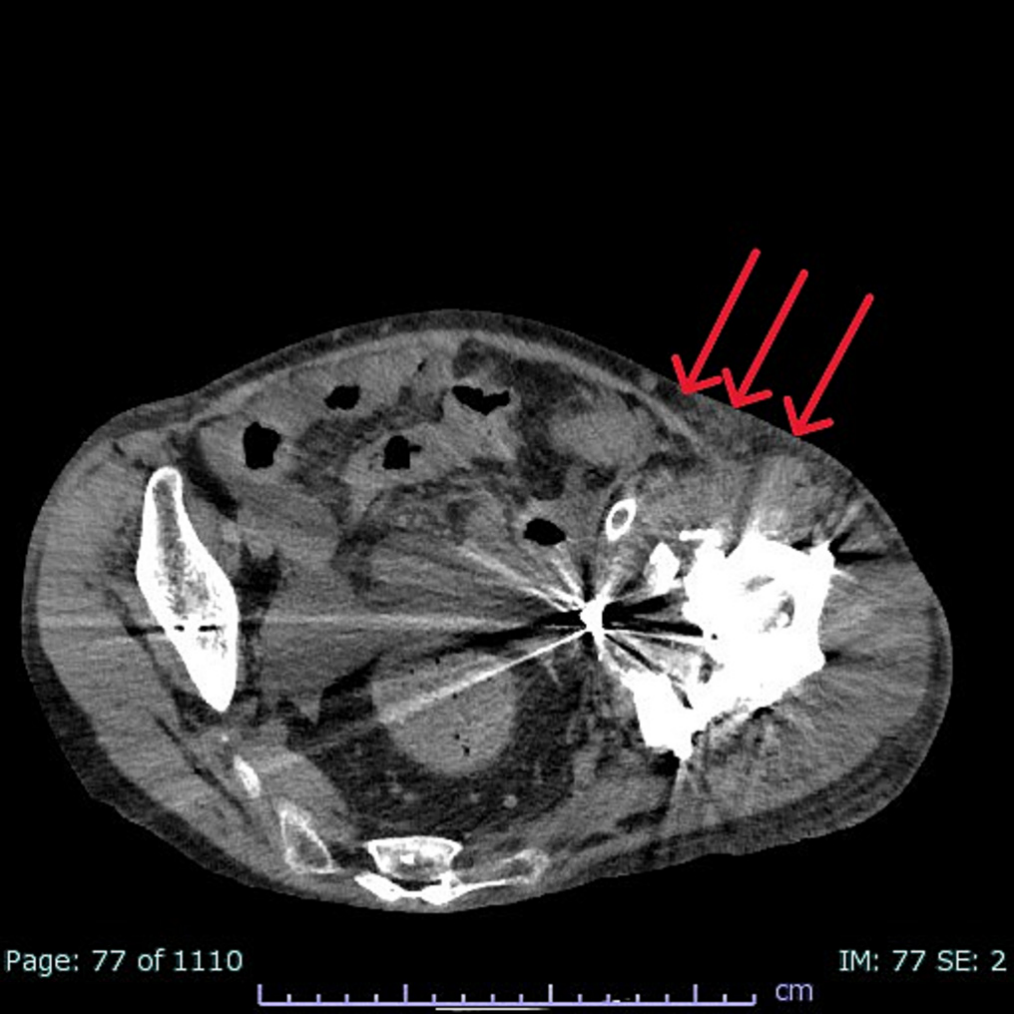
Follow-up computed tomography showed no re-expansion of the external iliac artery aneurysm or rupture, and the implanted stent graft was patent and well positioned.

After EVT in the cardiology department, the orthopedic surgeon removed the left artificial hip joint. Methicillin-susceptible *Staphylococcus aureus* was identified in blood culture. In addition to intravenous meropenem and linezolid, local administration of gentamicin (CLAP therapy) was continued. No clinical evidence of stent-graft infection was observed, and the patient's general condition stabilized after 14 weeks of antimicrobial therapy. Because the infection was controlled, she underwent revision THA. On postoperative day 6, follow-up CT indicated no re-expansion of EIA aneurysm, no rupture, and no clinical or radiological signs of infection (Fig. 2). On day 232 she was discharged without recurrence of infection, and was able to ambulate independently.

## Discussion

Herein we describe a case of ruptured infected EIA aneurysm following THA that was successfully treated with VIABAHN endoprosthesis implantation. Deep infections occurring after THA may spread to surrounding tissues. EIA mycotic aneurysm arising from contiguous infection of surrounding tissues, as observed in the current patient, is extremely rare [5]. Once the vessel wall became involved the infection caused the release of inflammatory cytokines, and rapidly spread to the deeper vascular layers, resulting in the formation of an aneurysm—a process that may have been facilitated by the patient's use of immunosuppressive medication [6].

Compared with unruptured aneurysms, open surgical repair of ruptured aneurysms carries a higher risk of perioperative mortality; therefore, endovascular treatment may be an effective alternative [7]. Although the optimal role of endovascular repair in infected aneurysms remains controversial, a recent systematic review has shown that in patients with mycotic aortic aneurysms, endovascular repair is associated with lower intraoperative and early mortality but a higher incidence of late sepsis and reintervention, with overall mid- to long-term survival comparable to that of open surgical repair [8]. In addition, Nakajima et al. reported favorable midterm outcomes in patients with infected aortic and iliac aneurysms treated by a combination of endovascular aneurysm repair and abscess drainage [9]. In the present case, the patient was hemodynamically stable with preserved blood pressure; however, laboratory tests showed markedly elevated inflammatory markers, indicating an active infectious process. Active bleeding from the EIA combined with infection of a prosthetic hip joint rendered open surgical repair in an already infected field particularly challenging and raised concern about post operative sepsis. EVT allowed rapid hemostasis with preservation of distal perfusion and reduced surgical trauma, and was therefore selected as the initial treatment strategy with close clinical and imaging follow-up. Furthermore, in this high-risk clinical setting, EVT was conceived as both a life-saving damage-control procedure and a potential bridging therapy to subsequent open reconstruction or graft explantation should infection progress or recur.

No clinical or laboratory signs of infection were evident at the time of discharge or at the latest follow-up visit. Lifelong suppressive oral antibiotic therapy with a culture-directed combination of clindamycin and rifampicin has been instituted in view of the potential for delayed infection, and prolonged clinical and imaging surveillance remains necessary. However, the optimal duration and regimen of antibiotic therapy after stent-graft placement in an infected field remain uncertain, and our strategy in this case was guided by the patient's immunosuppressed status and by previous reports advocating prolonged or lifelong suppressive therapy in similar settings [10]. Further accumulation of long-term outcome data in patients undergoing endovascular treatment for infected iliac aneurysms will be essential to refine the indications, follow-up protocols, and antibiotic strategies for this challenging condition.

In conclusion, above we have described a case of an infected EIA aneurysm that developed and ruptured after THA. The aneurysm was successfully treated with a combination of endovascular stent graft placement and antibacterial therapy. This strategy may be a viable treatment option in patients at high surgical risk.

## Consent

Written informed consent was obtained from the patient.

## Declaration of competing interest

The authors declare that there are no conflicts of interest.

## Appendix A Supplementary data

Video 1 First angiography. We confirmed extravasation from the external iliac artery. Video 1

Video 2 Final angiography. We successfully stopped the bleeding. Video 2

## References

[1] Shahait A.D., Chagas C., Hussein S., Bhat Z. (2019). Unique case of delayed external iliac artery pseudoaneurysm after a remote total hip arthroplasty. BMJ Case Rep.

[2] Fukuhara S., Kanki S., Daimon M., Shimada R., Ozawa H., Katsumata T. (2017). Pseudoaneurysm of the external iliac artery is a rare late complication after total hip arthroplasty. J Vasc Surg Cases Innov Tech.

[3] Chen J.L., Yang T.Y., Chuang P.Y., Huang T.W., Huang K.C. (2018). Pseudoaneurysm rupture with hemorrhagic shock in a patient with periprosthetic hip joint infection: a case report. Medicine (Baltimore).

[4] Bacharach J.M., Slovut D.P. (2008). State of the art: management of iliac artery aneurysmal disease. Catheter Cardiovasc Interv.

[5] Huang T.W., Wang C.J. (2004). Infected total knee arthroplasty complicated with mycotic aneurysms. A case report and review of the literature. Injury.

[6] Hall W.A., Majeed H., Ahmad F. (2025). StatPearls.

[7] IMPROVE Trial Investigators, Powell J.T., Sweeting M.J., Thompson M.M., Ashleigh R., Bell R., Gomes M., Greenhalgh R.M., Grieve R., Heatley F., Hinchliffe R.J., Thompson S.G., Ulug P. (2014). Endovascular or open repair strategy for ruptured abdominal aortic aneurysm: 30 day outcomes from IMPROVE randomised trial. BMJ.

[8] Li H.-L., Kwan K.J., Chan Y.C., Cheng S.W. (2024). Contemporary outcomes of endovascular and open surgical repair for mycotic aortic aneurysms: a systematic review. Ann Vasc Surg.

[9] Nakajima K., Kato N., Hashimoto T., Chino S., Higashigawa T., Ouchi T., Tokui T., Miyake Y., Sakuma H. (2018). Treatment of infected aneurysm with combined endovascular aneurysm repair and abscess drainage. J Vasc Interv Radiol.

[10] Hashimoto K., Isaka F., Yamashita K. (2015). An infected aneurysm of the vertebral artery treated with a stent-graft: a case report. Neurol Med Chir (Tokyo).

